# Comparative Analysis of Clinical Characteristics and Antimicrobial Resistance Between *Acinetobacter baumannii* and Other *Acinetobacter* Species

**DOI:** 10.3390/pathogens14010046

**Published:** 2025-01-08

**Authors:** Si-Ho Kim, Seok Jun Mun

**Affiliations:** 1 Division of Infectious Diseases, Department of Medicine, Samsung Changwon Hospital, Sungkyunkwan University School of Medicine, Changwon 51353, Republic of Korea; wychhazel@naver.com; 2 Division of Infectious Diseases, Department of Internal Medicine, Inje University Busan Paik Hospital, Inje University College of Medicine, Busan 47392, Republic of Korea; 3 Paik Institute for Clinical Research, Inje University College of Medicine, Busan 47392, Republic of Korea

**Keywords:** *Acinetobacter*, *Acinetobacter baumannii*, carbapenems, drug resistance, microbial, mortality

## Abstract

*Acinetobacter* species are major pathogens responsible for hospital-acquired infections. This study aimed to compare the clinical characteristics, outcomes, and antimicrobial resistance between *Acinetobacter baumannii* (AB) and non-*baumannii Acinetobacter* (NBA) species. In this retrospective cohort study, we analyzed data from adult patients (aged 18 or older) with *Acinetobacter* bacteremia treated at two tertiary hospitals from July 2020 to November 2023. Among 260 cases of *Acinetobacter* bacteremia, 42 (16.2%) involved NBA species. The AB group exhibited higher antimicrobial resistance rates across all tested agents, except for minocycline. Female patients, younger patients, and those with catheter-related infections were more commonly observed in the NBA group than in the AB group, while pneumonia and septic shock were more prevalent in the AB group. In a multivariable analysis of 30-day mortality, factors associated with higher mortality included moderate to severe liver disease, chronic kidney disease, carbapenem resistance, septic shock, and higher Pitt Bacteremia Scores. When stratified by carbapenem resistance (CR) status, only CR-AB exhibited significantly lower 30-day survival rate (33.4%) compared to that of the other groups (non-CR-NBA, 77.3%; CR-NBA, 77.8%; non-CR-AB, 90.1%; *p* < 0.001). Our findings highlight distinct clinical differences between AB and NBA bacteremia cases; however, the mortality rate for non-CR-AB was comparable to that observed in NBA bacteremia.

## 1. Introduction

*Acinetobacter*, a complex bacterial genus, is a common cause of hospital-acquired infections (HAI), particularly pneumonia and catheter-associated infections [1]. Among the various species within the genus, *Acinetobacter baumannii* has been reported as the most common species associated with infections and exhibits the highest virulence, accounting for 60–90% of *A. baumannii* bacteremia cases [1,2,3,4]. Close relatives of *A. baumannii*—*A. calcoaceticus*, *A. geminorum*, *A. lactucae* (formerly also known as *A. dijkshoorniae)*, *A. nosocomialis, A. oleivorans*, *A. pittii*, *and A. seifertii*—collectively form the *Acinetobacter calcoaceticus–baumannii (ACB)* complex. Although species within the ACB complex are nearly phenotypically indistinguishable, they exhibit substantial differences in ecology, pathogenicity, epidemiology, and antibiotic susceptibility [5]. These species also contribute to HAIs [1,4]. Additionally, other *Acinetobacter* species outside the ACB complex, such as *A. lwoffii*, have occasionally been reported as pathogens [1,6,7]. Since the adoption of matrix-assisted laser desorption ionization-time of flight (MALDI-TOF) mass spectrometry (MS) in clinical settings, *Acinetobacter* species have been reliably identified with over 97% accuracy [8,9]. Therefore, clinicians now have access to more accurate information on specific *Acinetobacter* species; however, only a few studies have described infections caused by species other than *A. baumannii.* Furthermore, no consensus has been established on the specific characteristics of infections caused by non-*baumannii Acinetobacter* (NBA) species in comparison to *A. baumannii* [2,3,4,10]. In this study, we describe the clinical characteristics and antimicrobial resistance profiles of bacteremia caused by NBA species and compare these to those of *A. baumannii* bacteremia.

## 2. Materials and Methods

### 2.1. Study Population, Definitions, and Design

This retrospective cohort study was conducted from July 2020 to November 2023 in two university-affiliated tertiary hospitals: Samsung Changwon Hospital (760 beds) and Inje University Busan Paik Hospital (818 beds). The inclusion criteria were adults (aged 18 or older) with *Acinetobacter* species bacteremia, which was isolated from one or more blood cultures. Patients were excluded if they (1) had polymicrobial bacteremia, (2) were lost to follow-up within 5 days, or (3) had *Acinetobacter* species isolated from only one blood culture bottle without any symptoms suggesting infection. Patient demographics and laboratory data were collected from electronic medical records. The updated Charlson Comorbidity Index was used to assess the degree of comorbidity, and the severity of bacteremia was evaluated using the Pitt Bacteremia Score (PBS) [11,12]. Intravenous catheter-related infection (CRI) is defined as an infection associated with peripheral intravascular catheters or central venous catheters [13]. The objectives of this study were to investigate the characteristics, antimicrobial resistance, and mortality of various *Acinetobacter* species. Therefore, the clinical characteristics and antimicrobial resistance were compared between *A. baumannii* and NBA species. Additionally, a subgroup analysis was conducted to compare the ACB complex (excluding *A. baumannii*) with the non-ACB complex within the NBA group. Patients included in this study were observed for 30 days after bacteremia, or until death or loss to follow-up. The survival rates of the *A. baumannii* and NBA groups were calculated, and factors associated with overall 30-day mortality were analyzed.

### 2.2. Microbiologic Methods

Blood samples were collected via a peripheral vein and/or central line, with two sets of blood culture bottles (aerobic and anaerobic, 8–10 cc each) prepared for incubation in either the Bactec-9240 system (Becton Dickinson, Sparks, MD, USA) or the BacT/Alert 3D system (bioMérieux, Marcy l’Etoile, France). All samples were cultured on blood agar and MacConkey agar plates in a 35 °C incubator for 24 h and identified by MALDI-TOF MS using the Vitek MS system (bioMérieux, Hazelwood, MI, USA). Antimicrobial susceptibility tests (ASTs) were conducted with the Vitek II automated system (bioMérieux, Marcy-l’Étoile, France). following the Clinical and Laboratory Standards Institute (CLSI) 2023 guidelines [14]. All procedures adhered to the manufacturers’ instructions.

### 2.3. Statistical Methods

The statistical analysis was conducted using IBM SPSS Statistics for Windows, version 25.0 (IBM Corp., 2018, Chicago, IL, USA), and R software, version 4.2.1 (R Foundation for Statistical Computing, Vienna, Austria). Continuous variables were analyzed with Student’s *t*-test or the Mann–Whitney U test, while categorical variables were compared using the chi-square test or Fisher’s exact test. The survival rate and curve were plotted using the Kaplan–Meier method and compared using the log-rank test. Risk factors for overall 30-day mortality were evaluated with a Cox regression model. Variables with *p* < 0.15 in the univariable analysis were included in the multivariable analysis using a forward stepwise Cox regression model. All *p*-values were two-tailed, and *p* < 0.05 was considered statistically significant.

### 2.4. Ethical Considerations

The study was approved by the Institutional Review Board of Samsung Medical Center (IRB file number: SCMC 2024-11-009) and Busan Paik Hospital (BPIRB 2024-11-024) with a waiver for informed consent. This waiver was granted as this was an observational retrospective study, and all patient data were analyzed anonymously.

## 3. Results

### 3.1. Study Population and Isolated Acinetobacter Species

**Figure 1:**
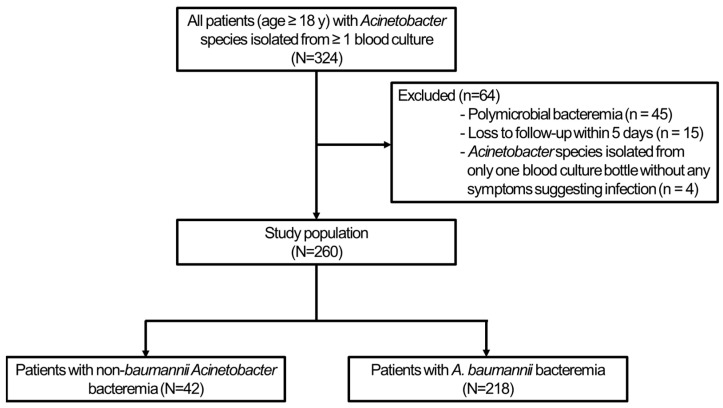
Study population.

**Figure 2:**
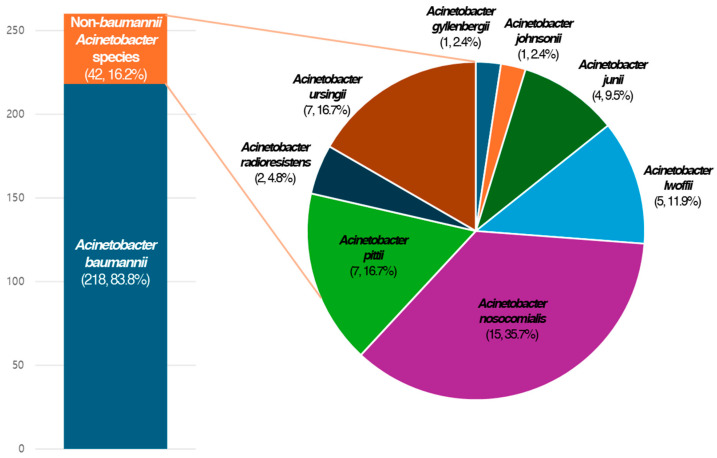
Distribution of isolated *Acinetobacter* species in the study population.

A total of 260 patients with *Acinetobacter* bacteremia were included in this study. Of the 260 cases, 42 (16.2%) involved the NBA species (Figure 1). Among the NBA species, 22 cases were of the ACB complex, comprising 15 cases of *A. nosocomialis* and 7 cases of *A. pittii*. Of the 20 cases of non-ACB complex, *A. ursingii* (7 cases) was the most common species, followed by *A. lwoffii* (5 cases, Figure 2).

### 3.2. Clinical Characteristics and Antimicrobial Resistances in Each Acinetobacter Species Group

In comparing patients with *A. baumannii* bacteremia to those with NBA, the former group had a higher prevalence of male sex, diabetes with end-organ damage, end-stage renal disease, and carbapenem resistance. Pneumonia was also more frequent, and disease severity was more pronounced in the *A. baumannii* bacteremia group than in the NBA group. In contrast, patients with NBA bacteremia were younger, had a higher incidence of intravenous CRI, and received appropriate antibiotics earlier in the course of infection compared to those with *A. baumannii* (Table 1). When comparing antimicrobial resistance between the groups, *A. baumannii* isolates showed higher resistance rates to all antibiotics except minocycline (Table 2). In the subgroup analysis comparing the ACB complex (excluding *A. baumannii*) with the non-ACB complex, no statistically significant differences were observed, except for carbapenem and gentamicin resistance rates, which were higher in the ACB complex group (Supplementary Tables S1 and S2).

### 3.3. Risk Factors for Overall 30-Day Mortality

**Figure 3:**
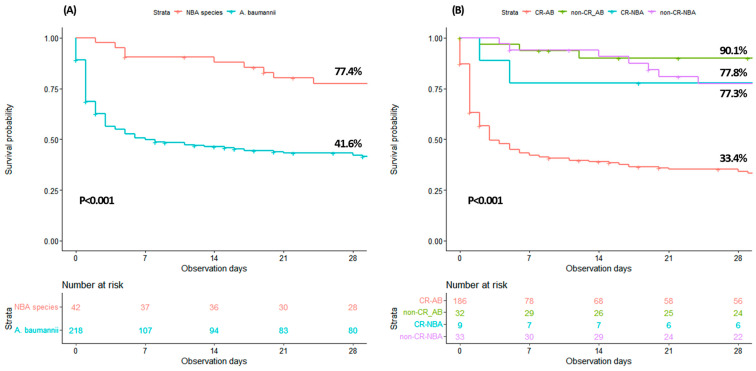
The survival curve illustrates outcomes among patients with different *Acinetobacter* species bacteremia (**A**) Comparison of survival between patients with *A. baumannii* bacteremia and NBA bacteremia. (**B**) Comparison of survival among patients with *Acinetobacter* bacteremia, stratified by species and carbapenem resistance status (post hoc analysis: CRAB vs. non-CRAB [*p* < 0.001], CRAB vs. CR-NAB [*p* = 0.041], CRAB vs. non-CR- NAB [*p* < 0.001]). NBA, non-*baumannii Acinetobacter*; CR, carbapenem resistance; AB, *A. baumannii*.

The overall 30-day survival rates were 41.6% for patients with *A. baumannii* bacteremia and 77.4% for those with NBA bacteremia (*p* < 0.0001 by log-rank test, Figure 3A). In contrast, no significant differences were observed between the ACB complex (excluding *A. baumannii*) and the non-ACB complex bacteremia, with survival rates of 81.3% and 72.7%, respectively (*p* = 0.564, Supplementary Figure S1). Supplementary Table S3 details factors associated with 30-day mortality in the study population based on a Cox regression model. In the multivariable analysis, moderate to severe liver disease (adjusted hazard ratio [aHR] 2.092, 95% CI 1.159–3.775, *p* = 0.014), moderate to severe renal disease (aHR 1.951, 95% CI 1.245–3.059, *p* = 0.004), carbapenem resistance (aHR 2.145, 95% CI 1.057–4.351, *p* = 0.035), septic shock (aHR 2.450, 95% CI 1.353–4.437, *p* = 0.003), and PBS (aHR 1.200, 95% CI 1.121–1.284, *p* < 0.001) were associated with higher mortality. Conversely, appropriate antibiotic therapy (aHR 0.219, 95% CI 0.138–0.349, *p* < 0.001) was associated with lower mortality (Table 3). Since carbapenem resistance was an independent factor associated with mortality, the stratification of patients with *Acinetobacter* bacteremia by carbapenem resistance (CR) status revealed that only the CR-AB group had a significantly lower 30-day survival rate (33.4%) compared to the other groups (non-CR-NBA, 77.3%; CR-NBA, 77.8%; non-CR-AB, 90.1%; *p* < 0.001, Figure 3B).

Supplementary Table S4 presents a subgroup analysis of factors associated with 30-day mortality in patients who received appropriate antimicrobial therapy, using a Cox regression model. In the multivariable analysis, moderate to severe renal disease (aHR 3.986, 95% CI 1.817–8.743, *p* = 0.001), pneumonia (aHR 2.466, 95% CI 1.099–5.534, *p* = 0.029), and PBS (aHR 1.382, 95% CI 1.174–1.627, *p* < 0.001) were associated with higher mortality (Table 4).

## 4. Discussion

Our study revealed that approximately one in six cases of *Acinetobacter* bacteremia was caused by NBA. The ratio of ACB complex to non-ACB species among NBA cases was close to 1:1. The characteristics and antimicrobial resistance profiles were significantly different between *A. baumannii* and NBA cases. However, among NBA cases, carbapenem and gentamicin resistance rates were the only significant differences observed between the ACB complex and non-ACB species. Regarding the 30-day survival rates, patients with *A. baumannii* bacteremia showed significantly lower survival rates compared to those in the NBA group; however, when stratified by carbapenem resistance, only patients with CRAB bacteremia exhibited a significantly lower survival rate compared to other groups.

The genus name *Acinetobacter*, derived from the Greek word ακινητος (akinetos), meaning “nonmotile”, was first introduced in 1954 to distinguish nonmotile microorganisms from motile ones within the genus *Achromobacter* [15]. Subsequently, Baumann et al. conducted a comprehensive study and concluded that the various species mentioned above belonged to a single genus, which they proposed naming *Acinetobacter*; the group also reported that further subclassification into distinct species based on phenotypic characteristics was not feasible [16]. A significant advancement in the complex history of the *Acinetobacter* genus occurred in 1986 when Bouvet and Grimont used DNA–DNA hybridization studies to identify 12 DNA hybridization groups (or genospecies). Some of these groups were assigned formal species names, including *A. baumannii* [17]. Although species within *Acinetobacter* are difficult to classify based on phenotype, there are differences in the clinical characteristics and antimicrobial resistance patterns between *A. baumannii* and NBA [1]. First, *A. baumannii* has a higher virulence than do NBA species [1]. For example, *A. baumannii* strains demonstrated higher lethality toward wax moth larvae (*Galleria mellonella*) compared to strains of *A. baylyi* and *A. lwoffii* [18]. A study conducted in the United States, which analyzed *Acinetobacter* blood isolates collected between 1995 and 2003 from 52 hospitals, reported that *A. baumannii* (187/295) was the most prevalent species, followed by *A. nosocomialis* (61/295), *A. pittii* (23/295), and other NBA species (24/295). The mortality rates associated with these groups were 36.9%, 16.4%, 13.0%, and 4.2%, respectively, with statistically significant differences observed among the groups (*p* < 0.001) [2]. Second, both NBA species and *A. baumannii* are commonly found in wet environments and hospital settings. NBA species are frequently isolated from the skin of healthy individuals, whereas *A. baumannii* is rarely found on healthy skin and is primarily associated with hospital environments and immunocompromised patients [19,20]. In clinical studies involving hospitalized patients, those with NBA bacteremia were found to have fewer underlying conditions and lower disease severity than those with AB bacteremia. A study conducted in Korea further reported that patients with NBA bacteremia had lower rates of ICU admission (14.3% in the NBA group vs. 49.1% in the *A. baumannii* group, *p* < 0.001) and utilization of indwelling Foley catheters (21.4% vs. 60.7%, *p* < 0.001) and mechanical ventilation (3.6% vs. 40.2%, *p* < 0.001) [10]. Another study conducted on Korean children also revealed that malignancy was more common among patients infected with the ACB complex than it was in the non-ACB complex group [4]. Third, *A. baumannii* exhibited higher levels of antimicrobial resistance compared to the NBA species. While antimicrobial resistance varies greatly by geographic region, carbapenem resistance in *A. baumannii* has been reported to exceed 50% in Southern and Eastern Europe, reaching approximately 90% in the Republic of Korea [21,22]. However, carbapenem resistance in NBA species was reported to be approximately 10% or lower [2,3,4,22,23].

Our study findings were consistent with those reported in previous studies. The 30-day mortality rate of patients with *A. baumannii* bacteremia was more than two times higher than that of patients with NBA bacteremia. The most common portals of entry for bacteremia in each group were pneumonia and CRI, respectively. In addition, patients with *A. baumannii* bacteremia had higher rates of diabetes mellitus with end-organ damage and moderate to severe renal disease than those with NBA bacteremia. Furthermore, more patients with *A. baumannii* bacteremia had septic shock and had higher PBS than those with NBA bacteremia. Except for minocycline, resistance to all other antimicrobials was higher in *A. baumannii* isolates than in NBA isolates. The novelty of our study is that we classified *Acinetobacter* species not only into *A. baumannii* and NBA but also into ACB complex and non-ACB complex. Previous studies primarily compared the characteristics of various NBA species with *A. baumannii* [2,3,7] or overall non-ACB complex species with the ACB complex [4,10]. In contrast, our study included a subgroup analysis comparing the ACB complex (excluding *A. baumannii*) with the non-ACB complex, which revealed no significant differences in patient characteristics, clinical manifestations, or outcomes. However, carbapenem and gentamicin resistances were notably more common in the ACB complex group than in the NBA group. It is known that NBA species can exhibit carbapenem resistance [4,23,24], which is associated with beta-lactamase genes. However, to the best of our knowledge, this is the first study to report distinct antimicrobial resistance patterns between these two groups. In addition, analysis of factors associated with mortality revealed that carbapenem resistance was independently associated with higher mortality, regardless of whether the infection was caused by *A. baumannii* or NBA. This differs from a previous report from Thailand, which suggested that infection with CR-AB was associated with 30-day mortality (adjusted OR 2.50, 95% CI 1.03–6.25, *p* = 0.029), whereas infection with non-ACB complex species was associated with lower 30-day mortality (adjusted OR 0.09, 95% CI 0.02–0.41, *p* = 0.005) [3]. Therefore, further studies are needed to evaluate the contributions of specific *Acinetobacter* species and carbapenem resistance to patient outcomes.

Our study has several limitations. First, *Acinetobacter* species were identified using MALDI-TOF MS. Although MALDI-TOF MS demonstrates high accuracy (exceeding 97%) in identifying *Acinetobacter* species, including non-*A. baumannii* members of the ACB complex, the gold standard for species identification remains 16S rDNA and rpoB gene sequencing [8]. Second, this study was conducted in two medical centers in the Republic of Korea. Antimicrobial resistance patterns can vary significantly by period and geographic location. For example, in settings where the carbapenem resistance rate of *A. baumannii* is low, there might be no significant difference between *A. baumannii* and NBA isolates. However, even in the absence of differences in carbapenem resistance, other antimicrobial resistance tended to be higher in *A. baumannii* isolates [2,10]. Third, the disparity in sample sizes between the *A. baumannii* and NBA groups (particularly for carbapenem resistance in the NBA group) may have limited our ability to detect certain differences. Specifically, the relatively small sample size of the NBA group could have reduced the statistical power of the analysis.

## 5. Conclusions

In conclusion, our study suggested that a significant portion of NBA species contribute to clinical infections among *Acinetobacter* species. In addition to *A. baumannii*, other species within the ACB complex should also be monitored for antimicrobial resistance. Although patients with *A. baumannii* bacteremia exhibited higher mortality than those with NBA bacteremia, this difference may be attributable to carbapenem resistance rather than to the intrinsic virulence of *A. baumannii*. Further large-scale and prospective studies are needed to evaluate the clinical characteristics and burden of infections caused by NBA species.

## Figures and Tables

**Table 1:** Clinical characteristics of patient with *Acinetobacter* bacteremia.

Characteristics	Overall *(n* = 260)	Non-*baumannii Acinetobacter* Bacteremia (*n* = 42)	*A. baumannii* Bacteremia (*n* = 218)	*p* Value
Male	154 (59.2)	17 (40.5)	137 (62.8)	0.007
Age, years, median (IQR)	70 (61.25–77)	65.5 (51–75)	71 (63–77)	0.031
Comorbidities				
Updated CCI, median (IQR)	2 (1–4)	2 (1–5)	2 (1–4)	0.717
Hypertension	114 (43.8)	22 (52.4)	92 (42.2)	0.224
DM with end-organ damage	43 (16.5)	2 (4.8)	41 (18.8)	0.025
Moderate to severe liver disease	17 (6.5)	1 (2.4)	16 (7.3)	0.323
Moderate to severe renal disease	33 (12.7)	1 (2.4)	32 (14.7)	0.028
Metastatic solid tumor	37 (14.2)	8 (19.1)	29 (13.3)	0.329
Carbapenem resistance	195 (75.0)	9 (21.4)	186 (85.3)	<0.001
Focus of bacteremia				
Primary bacteremia	26 (10)	7 (16.7)	19 (8.7)	0.155
Intravascular catheter-related infection	78 (30.0)	29 (69.1)	49 (22.5)	<0.001
Pneumonia	126 (48.5)	2 (4.8)	124 (56.9)	<0.001
Intra-abdominal infections	12 (4.6)	2 (4.8)	10 (4.6)	>0.999
SSTI	5 (1.9)	0 (0.0)	5 (2.3)	>0.999
UTI	10 (3.8)	2 (4.8)	8 (3.7)	0.667
Other infections	4 (1.5)	0 (0.0)	4 (1.8)	>0.999
Initial severity				
Septic shock	144 (55.4)	17 (40.5)	127 (58.3)	0.034
Pitt bacteremia score	4 (1–7)	2 (1–3)	5 (1–8)	0.002
Renal replacement therapy	44 (16.9)	4 (9.5)	40 (18.4)	0.163
Antibiotic therapy				
AAT	146 (56.2)	37 (88.1)	109 (50.0)	<0.001
Time to AAT, days, median (IQR)	1 (0–3)	1 (0–2)	2 (0–3)	0.009
Colistin containing regimen (*n* = 146)	75 (51.4)	7 (18.9)	68 (62.4)	<0.001
Tetracycline containing regimen (*n* = 146)	46 (31.5)	3 (8.1)	43 (39.5)	0.004
Carbapenem containing regimen (*n* = 146)	71 (48.6)	21 (56.8)	50 (45.9)	0.252
Ampicillin-sulbactam containing regimen (*n* = 146)	11 (7.5)	3 (8.1)	8 (7.3)	>0.999
Combination therapy ^a^ (*n* = 146)	31 (21.2)	8 (21.6)	23 (21.1)	0.947

Data are presented as numbers (%) unless otherwise indicated. ^a^ Combination therapy was defined as the administration of two or more effective antibiotics. Abbreviations: AAT, appropriate antibiotic therapy; CCI, Charlson Comorbidity Index; CKD, chronic kidney disease; CRI, catheter-related infections; DM, diabetes mellitus; IQR, interquartile range; SSTI, skin and soft tissue infections; UTI, urinary tract infections.

**Table 2:** Antibiotic resistance rate of *Acinetobacter* isolates.

Antibiotics	Non-*baumannii Acinetobacter* Species (*n* = 42)	*A. baumannii* (*n* = 218)	*p* Value
Ampicillin-sulbactam	5 (11.9)	169 (77.5)	<0.001
Ceftazidime	20 (47.6)	192 (88.1)	<0.001
Piperacillin-tazobactam	12 (28.6)	190 (87.2)	<0.001
Meropenem	9 (21.4)	186 (85.3)	<0.001
Imipenem	8 (19.1)	185 (84.9)	<0.001
Minocycline	0 (0.0)	10 (4.6)	0.377
Trimethoprim-sulfamethoxazole	3 (7.1)	157 (72.0)	<0.001
Gentamicin	12 (28.6)	145 (66.5)	<0.001

Data are presented as numbers (%).

**Table 3:** Univariable and multivariable analyses for overall 30-day mortality in patients with *Acinetobacter* bacteremia.

Variable	HR (95% CI)	*p* Value	Adjusted HR (95% CI)	*p* Value
*A. baumannii* bacteremia	3.707 (1.882–7.302)	<0.001		
Male	1.429 (0.999–2.043)	0.051		
Age (per 1 year)	1.016 (1.001–1.031)	0.031		
DM with end-organ damage	1.775 (1.186–2.658)	0.005		
Moderate to severe liver disease	2.265 (1.276–4.019)	0.005	2.092 (1.159–3.775)	0.014
Moderate to severe renal disease	1.958 (1.273–3.011)	0.002	1.951 (1.245–3.059)	0.004
Carbapenem resistance	6.075 (3.182–11.597)	<0.001	2.145 (1.057–4.351)	0.035
Intravascular CRI	0.405 (0.260–0.630)	<0.001		
Pneumonia	3.822 (2.617–5.582)	<0.001		
Intra-abdominal infections	0.276 (0.068–1.116)	0.071		
Urinary tract infection	0.294 (0.073–1.186)	0.085		
Septic shock	7.684 (4.820–12.249)	<0.001	2.450 (1.353–4.437)	0.003
PBS (per 1 point)	1.383 (1.312–1.457)	<0.001	1.200 (1.121–1.284)	<0.001
Renal replacement therapy	2.486 (1.693–3.649)	<0.001		
AAT	0.131 (0.089–0.194)	<0.001	0.219 (0.138–0.349)	<0.001

HR, hazard ratio; DM, diabetes mellitus; CI, confidence interval; CRI, catheter-related infections; PBS, Pitt Bacteremia Score; AAT, appropriate antibiotic therapy.

**Table 4:** Univariable and multivariable analyses for 30-day in-hospital mortality in patients with *Acinetobacter* bacteremia who received appropriate antibiotic therapy.

Variable	HR (95% CI)	*p* Value	Adjusted HR (95% CI)	*p* Value
DM with end-organ damage	2.674 (1.334–5.362)	0.006		
Moderate to severe CKD	2.989 (1.420–6.291)	0.004	3.986 (1.817–8.743)	0.001
Carbapenem resistance	1.950 (0.953–3.989)	0.068		
Intravascular CRI	0.267 (0.118–0.603)	0.002		
Pneumonia	5.600 (2.838–11.050)	<0.001	2.466 (1.099–5.534)	0.029
Septic shock	4.739 (2.464–9.116)	<0.001		
PBS (per 1 point)	1.472 (1.287–1.683)	<0.001	1.382 (1.174–1.627)	<0.001
Renal replacement therapy	5.278 (2.725–10.225)	<0.001	c	
Colistin containing regimen	2.270 (1.170–4.404)	0.015		
Carbapenem containing regimen	2.213 (1.155–4.241)	0.017		

CCI, Charlson Comorbidity Index; CI, confidence interval; CKD, chronic kidney disease; CRI, catheter-related infections; DM, diabetes mellitus; HR, hazard ratio; PBS, Pitt Bacteremia Score.

## Data Availability

The raw data supporting the conclusions of this article will be made available by the authors upon request.

## Supplementary Materials

The following supporting information can be downloaded at: https://www.mdpi.com/article/10.3390/pathogens14010046/s1, Supplementary Table S1. Clinical characteristics of patients with ACB complex (excluding *A. baumannii*) and non-ACB complex bacteremia. Supplementary Table S2. Antibiotic resistance rate of ACB complex (excluding *A. baumannii*) and non-ACB complex bacteremia. Supplementary Table S3. Univariable analyses for overall 30-day in-hospital mortality in patients with *Acinetobacter* bacteremia. Supplementary Table S4. Univariable analyses for overall 30-day mortality in patients with *Acinetobacter* bacteremia who received appropriate antibiotic therapy. Supplementary Figure S1. The survival curve illustrates outcomes among patients with ACB complex (excluding *A. baumannii*) and non-ACB complex bacteremia.
