# Supplementary material for: Comparative Analysis of Clinical Characteristics and Antimicrobial Resistance Between Acinetobacter baumannii and Other Acinetobacter Species
Source: Pathogens. 2025 Jan 8;14(1):46. doi: 10.3390/pathogens14010046 (PMC11768325; doi:10.3390/pathogens14010046)
Supplement: Supplementary file 1 [file pathogens-14-00046-s001.zip › pathogens-3373258-supplementary.pdf]

Comparative Analysis of Clinical Characteristics and Antimicrobial Resistance Between *Acinetobacter baumannii* and Other *Acinetobacter* Species

SUPPLEMENTARY MATERIALS

Table S1. Clinical characteristics of patient with ACB complex (excluding *A. baumannii*) and non-ACB complex.

Table S2. Antibiotic resistance rate of ACB complex (excluding *A. baumannii*) and non-ACB complex.

table S3. Univariable analyses for overall 30-day in-hospital mortality in patients with *Acinetobacter* bacteremia

table S4. Univariable analyses for overall 30-day mortality in patients with *Acinetobacter* bacteremia who received appropriate antibiotic therapy

figure S1. The survival curve illustrates outcomes among patients with ACB complex (excluding *A. baumannii*) and non-ACB complex bacteremia

Table S1. Clinical characteristics of patient with ACB complex (excluding *A. baumannii)* and non-ACB complex.

Characteristics	non-*baumannii* ACB complex (n = 22)	non-ACB complex bacteremia (n = 20)	*P* value
Male	6 (27.3)	11 (55.0)	0.067
Age, years, median (IQR)	68 (53.5-75.0)	65 (51-76.5)	0.879
Comorbidities			
Updated CCI, median (IQR)	2 (0.75-6)	2 (1.25-3)	0.481
Hypertension	13 (59.1)	9 (45.0)	0.361
DM with end-organ damage	1 (4.5)	1 (5.0)	>0.999
Moderate to severe liver disease	1 (4.50)	0 (0.0)	>0.999
Moderate to severe CKD	0 (0.0)	1 (5.0)	0.476
Metastatic solid tumor	6 (27.3)	2 (10.0)	0.243
Carbapenem resistance	8 (36.4)	1 (5.0)	0.022
Focus of bacteremia			
Primary bacteremia	4 (18.2)	3 (15.0)	>0.999
Intravascular CRI	15 (68.2)	14 (70.0)	0.899
Pneumonia	1 (4.5)	1 (5.0)	>0.999
Intra-abdominal infections	1 (4.5)	1 (5.0)	>0.999
SSTI	0 (0.0)	0 (0.0)	>0.999
UTI	1 (4.5)	1 (5.0)	>0.999
Other infections	0 (0.0)	0 (0.0)	>0.999
Initial severity			
Septic shock	10 (45.5)	7 (35.0)	0.543
Pitt bacteremia score	2 (0.75-3)	2.5 (0.25-5.75)	0.369
Renal replacement therapy	2 (9.1)	2 (10.0)	>0.999
Antibiotic therapy			
AAT	18 (81.8)	19 (95.0)	0.346
Time to AAT, days, median (IQR)	0 (0-1.25)	0.5 (0-1.75)	
Colistin containing regimen (n = 37)	3 (16.7)	4 (21.1)	>0.999
Tetracyclines containing regimen (n = 37)	1 (5.6)	2 (10.5)	>0.999
Carbapenem containing regimen (n = 37)	9 (50.0)	12 (63.2)	0.515
Ampicillin-sulbactam containing regimen (n = 37)	3 (16.7)	0 (0.0)	0.105
Combination therapy[a] (n = 37)	3 (16.7)	5 (26.3)	0.693
30-day in-hospital mortality	4 (17.2)	5 (25.0)	0.714

Data are presented as the numbers (%) unless otherwise indicated.

Abbreviations: AAT, appropriate antibiotic therapy; CCI, Charlson Comorbidity Index; CKD, chronic kidney disease; CRI, catheter-related infections; DM, diabetes mellitus; IQR, interquartile range; SSTI, skin and soft tissue infections; UTI, urinary tract infections.

[a]Combination therapy was defined as the administration of two or more effective antibiotics.

Table S2. Antibiotic resistance rate of ACB complex (excluding *A. baumannii*) *and non-ACB complex.*

Antibiotics	non-*baumannii* ACB complex (n = 22)	non-ACB complex bacteremia (n = 20)	*P* value
Ampicillin-sulbactam	3 (13.6)	2 (10.0)	>0.999
Ceftazidime	11 (50.0)	9 (45.0)	0.746
Piperacillin-tazobactam	8 (36.4)	4 (20.0)	0.241
Meropenem	8 (36.4)	1 (5.0)	0.022
Imipenem	7 (31.8)	1 (5.0)	0.047
Minocycline	0 (0.0)	0 (0.0)	>0.999
Trimethoprim-sulfamethoxazole	1 (4.5)	2 (7.1)	0.598
Gentamicin	10 (45.5)	2 (10.0)	0.011

Data are presented as the numbers (%)

Table S3. Univariable analyses for overall 30-day in-hospital mortality in patients with *Acinetobacter* bacteremia

**Variable**	**HR (95% CI)**	***P* value**
Acinetobacter baumannii bacteremia	3.707 (1.882-7.302)	<0.001
Male	1.429 (0.999–2.043)	0.051
Age (per 1 year)	1.016 (1.001–1.031)	0.031
Comorbidities		
Updated CCI (per 1 point)	1.044 (0.973–1.120)	0.231
Hypertension	1.007 (0.715–1.418)	0.969
DM with end-organ damage	1.775 (1.186–2.658)	0.005
Moderate to severe liver disease	2.265 (1.276–4.019)	0.005
Moderate to severe CKD	1.958 (1.273–3.011)	0.002
Metastatic solid tumor	0.933(0.568–1.534)	0.785
Carbapenem resistance	6.075 (3.182–11.597)	<.001
Focus of bacteremia		
Primary bacteremia	0.658 (0.345–1.254)	0.203
Intravascular CRI	0.405 (0.260–0.630)	<.001
Pneumonia	3.822 (2.617–5.582)	<.001
Intra-abdominal infections	0.276 (0.068–1.116)	0.071
SSTI	0.279 (0.039–1.997)	0.204
UTI	0.294 (0.073–1.186)	0.085
Other infections	0.181 (0.001–1.238)	0.230
Initial severity		
Septic shock	7.684 (4.820–12.249)	<.001
PBS (per 1 point)	1.383 (1.312–1.457)	<.001
Renal replacement therapy	2.486 (1.693–3.649)	<.001
AAT	0.131 (0.089-0.194)	<.001

Abbreviations: AAT, appropriate antibiotic therapy; CCI, Charlson Comorbidity Index; CI, Confidence interval; CKD, chronic kidney disease; CRI, catheter-related infections; DM, diabetes mellitus; HR, hazard ratio; PBS, Pitt bacteremia score; SSTI, skin and soft tissue infections; UTI, urinary tract infections.

Table S4. Univariable analyses for overall 30-day mortality in patients with *Acinetobacter* bacteremia who received appropriate antibiotic therapy

Variable	HR (95% CI)	*P* value
Acinetobacter baumannii bacteremia	1.778 (0.786-4.020)	0.167
Male	1.080 (0.579–2.014)	0.809
Age (per 1 year)	1.011 (0.985–1.039)	0.411
Comorbidities		
Updated CCI (per 1 point)	1.056 (0.928–1.203)	0.407
Hypertension	1.228 (0.660–2.283)	0.516
DM with end-organ damage	2.674 (1.334–5.362)	0.005
Moderate to severe liver disease	1.212 (0.292–5.025)	0.791
Moderate to severe CKD	2.989 (1.420–6.291)	0.004
Metastatic solid tumor	0.936 (0.367–2.389)	0.890
Carbapenem resistance	1.950 (0.953–3.989)	0.068
Focus of bacteremia		
Primary bacteremia	0.694 (0.214–2.250)	0.542
Intravascular CRI	0.267 (0.118–0.603)	0.002
Pneumonia	5.600 (2.838–11.050)	<.001
Intra-abdominal infections	0.202 (0.002–1.418)	0.268
SSTI	0.620 (0.085–4.515)	0.637
UTI	0.487 (0.067–3.548)	0.478
Other infections	0.740 (0.006–5.183)	0.835
Initial severity		
Septic shock	4.739 (2.464–9.116)	<.001
PBS (per 1 point)	1.472 (1.287–1.683)	<.001
Renal replacement therapy	5.278 (2.725–10.225)	<.001
AAT		
Time between diagnosis to AAT (per day)	0.896 (0.757-1.059)	0.198
Colistin containing regimen	2.270 (1.170–4.404)	0.015
Tetracyclines containing regimen	1.001 (0.517–1.941)	0.997
Carbapenem containing regimen	2.213 (1.155–4.241)	0.017
Ampicillin-sulbactam containing regimen	0.631 (0.152–2.614)	0.525
Combination therapy	1.364 (0.681–2.731)	0.381

Abbreviations: AAT, appropriate antibiotic therapy; CCI, Charlson Comorbidity Index; CI, Confidence interval; CKD, chronic kidney disease; CRI, catheter-related infections; DM, diabetes mellitus; HR, hazard ratio; PBS, Pitt bacteremia score; SSTI, skin and soft tissue infections; UTI, urinary tract infections.

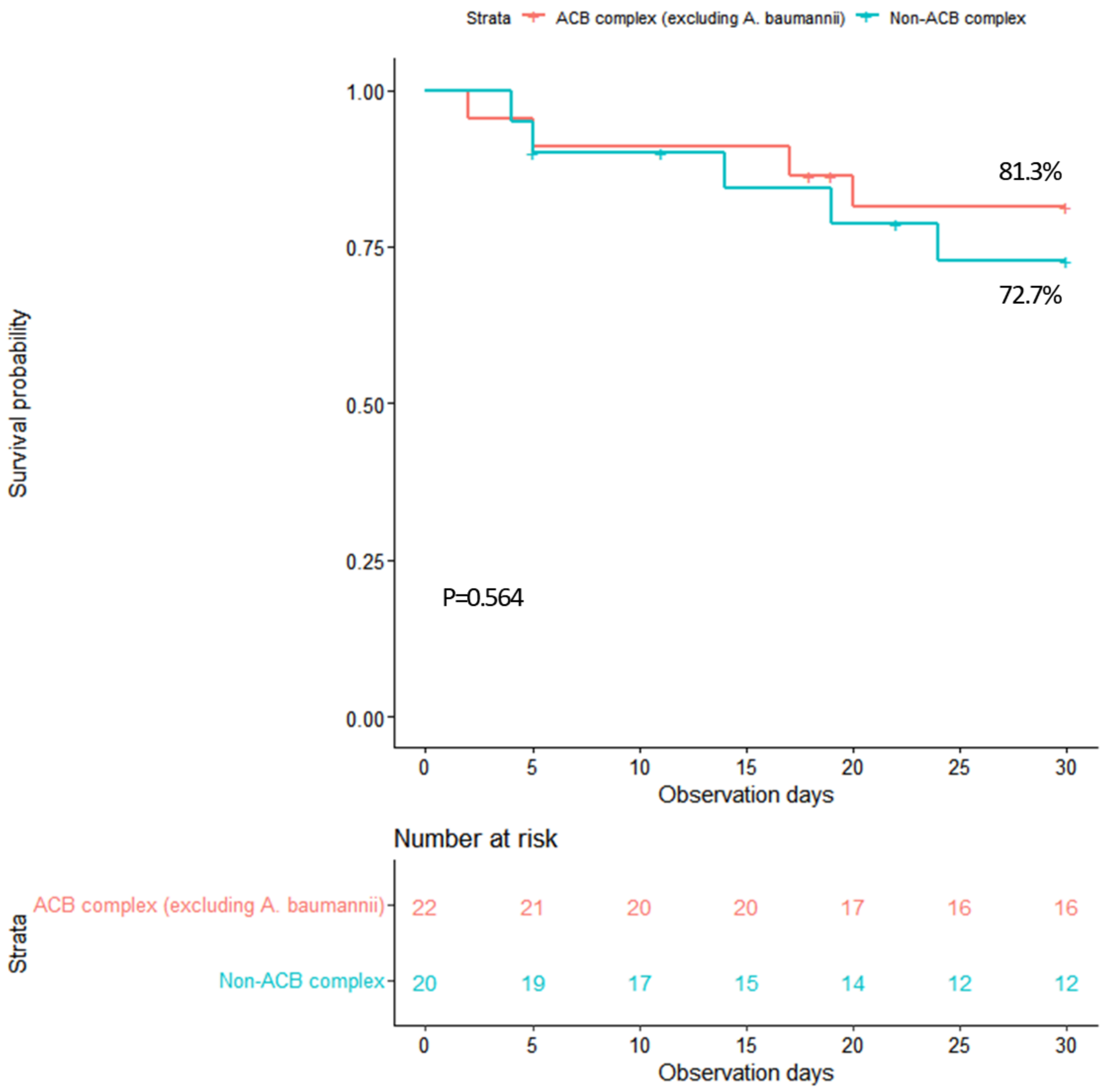

Figure S1. The survival curve illustrates outcomes among patients with *Acinetobacter calcoaceticus–baumannii (ACB)* complex (excluding *A. baumannii*) and non-ACB complex bacteremia
